# External validation of models for predicting cumulative live birth over multiple complete cycles of IVF treatment

**DOI:** 10.1093/humrep/dead165

**Published:** 2023-08-25

**Authors:** Mariam B Ratna, Siladitya Bhattacharya, David J McLernon

**Affiliations:** Institute of Applied Health Sciences, School of Medicine, Medical Sciences & Nutrition, University of Aberdeen, Aberdeen, UK; Clinical Trials Unit, Warwick Medical School, University of Warwick, Warwick, UK; School of Medicine, Medical Sciences & Nutrition, University of Aberdeen, Aberdeen, UK; Institute of Applied Health Sciences, School of Medicine, Medical Sciences & Nutrition, University of Aberdeen, Aberdeen, UK

**Keywords:** IVF, live birth, clinical prediction model, validation, calibration

## Abstract

**STUDY QUESTION:**

Can two prediction models developed using data from 1999 to 2009 accurately predict the cumulative probability of live birth per woman over multiple complete cycles of IVF in an updated UK cohort?

**SUMMARY ANSWER:**

After being updated, the models were able to estimate individualized chances of cumulative live birth over multiple complete cycles of IVF with greater accuracy.

**WHAT IS KNOWN ALREADY:**

The McLernon models were the first to predict cumulative live birth over multiple complete cycles of IVF. They were converted into an online calculator called OPIS (Outcome Prediction In Subfertility) which has 3000 users per month on average. A previous study externally validated the McLernon models using a Dutch prospective cohort containing data from 2011 to 2014. With changes in IVF practice over time, it is important that the McLernon models are externally validated on a more recent cohort of patients to ensure that predictions remain accurate.

**STUDY DESIGN, SIZE, DURATION:**

A population-based cohort of 91 035 women undergoing IVF in the UK between January 2010 and December 2016 was used for external validation. Data on frozen embryo transfers associated with these complete IVF cycles conducted from 1 January 2017 to 31 December 2017 were also collected.

**PARTICIPANTS/MATERIALS, SETTING, METHODS:**

Data on IVF treatments were obtained from the Human Fertilisation and Embryology Authority (HFEA). The predictive performances of the McLernon models were evaluated in terms of discrimination and calibration. Discrimination was assessed using the c-statistic and calibration was assessed using calibration-in-the-large, calibration slope, and calibration plots. Where any model demonstrated poor calibration in the validation cohort, the models were updated using intercept recalibration, logistic recalibration, or model revision to improve model performance.

**MAIN RESULTS AND THE ROLE OF CHANCE:**

Following exclusions, 91 035 women who underwent 144 734 complete cycles were included. The validation cohort had a similar distribution age profile to women in the development cohort. Live birth rates over all complete cycles of IVF per woman were higher in the validation cohort. After calibration assessment, both models required updating. The coefficients of the pre-treatment model were revised, and the updated model showed reasonable discrimination (c-statistic: 0.67, 95% CI: 0.66 to 0.68). After logistic recalibration, the post-treatment model showed good discrimination (c-statistic: 0.75, 95% CI: 0.74 to 0.76). As an example, in the updated pre-treatment model, a 30-year-old woman with 2 years of primary infertility has a 41% chance of having a live birth in the first complete ICSI cycle and a 75% chance over three complete cycles. In a couple with 2 years of primary male factor infertility where a 30-year-old woman has 15 oocytes collected in the first cycle, a single fresh blastocyst embryo transferred in the first cycle and spare embryos cryopreserved, the estimated chance of live birth provided by the post-treatment model is 40% in the first complete ICSI cycle and 75% over three complete cycles.

**LIMITATIONS, REASONS FOR CAUTION:**

Two predictors from the original models, duration of infertility and previous pregnancy, which were not available in the recent HFEA dataset, were imputed using data from the older cohort used to develop the models. The HFEA dataset does not contain some other potentially important predictors, e.g. BMI, ethnicity, race, smoking and alcohol intake in women, as well as measures of ovarian reserve such as antral follicle count.

**WIDER IMPLICATIONS OF THE FINDINGS:**

Both updated models show improved predictive ability and provide estimates which are more reflective of current practice and patient case mix. The updated OPIS tool can be used by clinicians to help shape couples’ expectations by informing them of their individualized chances of live birth over a sequence of multiple complete cycles of IVF.

**STUDY FUNDING/COMPETING INTEREST(S):**

This study was supported by an Elphinstone scholarship scheme at the University of Aberdeen and Aberdeen Fertility Centre, University of Aberdeen. S.B. has a commitment of research funding from Merck. D.J.M. and M.B.R. declare support for the present manuscript from Elphinstone scholarship scheme at the University of Aberdeen and Assisted Reproduction Unit at Aberdeen Fertility Centre, University of Aberdeen. D.J.M. declares grants received by University of Aberdeen from NHS Grampian, The Meikle Foundation, and Chief Scientist Office in the past 3 years. D.J.M. declares receiving an honorarium for lectures from Merck. D.J.M. is Associate Editor of *Human Reproduction Open* and Statistical Advisor for *Reproductive BioMed Online.* S.B. declares royalties from Cambridge University Press for a book. S.B. declares receiving an honorarium for lectures from Merck, Organon, Ferring, Obstetric and Gynaecological Society of Singapore, and Taiwanese Society for Reproductive Medicine. S.B. has received support from Merck, ESHRE, and Ferring for attending meetings as speaker and is on the METAFOR and CAPRE Trials Data Monitoring Committee.

**TRIAL REGISTRATION NUMBER:**

N/A.

## Introduction

A recent systematic review identified over 30 clinical prediction models which estimate individualized chances of pregnancy outcomes following IVF treatment (Ratna *et al.*, 2020). These models can help clinicians communicate chances of treatment success to couples undergoing IVF, but their use in clinical practice has been limited. The quality of these models is impacted by issues such as small sample sizes, lack of external validation and failure to demonstrate clinical impact (Leushuis *et al.*, 2009; Van Loendersloot *et al.*, 2014; Ratna *et al.*, 2020).

Five IVF prediction model studies have been conducted using large national databases (Templeton *et al.*, 1996; Nelson and Lawlor, 2011; Luke *et al.*, 2014; McLernon *et al.*, 2016; McLernon *et al.*, 2021). Of these, three utilized data from the Human Fertilisation and Embryology Authority (HFEA) registry in the UK to estimate the chances of a live birth after IVF (Templeton *et al.*, 1996; Nelson and Lawlor, 2011; McLernon *et al.*, 2016). Two of these articles published models that predict cumulative live birth over complete cycles of IVF, where a complete cycle is defined as all fresh and frozen-thawed embryo transfers associated with one episode of ovarian stimulation (McLernon *et al.*, 2016; McLernon *et al.*, 2021). With the increasing use of frozen-thawed embryos in IVF (Wong *et al.*, 2014), cumulative live birth rate (LBR) over multiple complete cycles is a more clinically relevant outcome than the chance of live birth following a single embryo transfer (Maheshwari *et al.*, 2015) and clinical prediction models need to make sure that they address this need (McLernon and Bhattacharya, 2023).

Two UK models by McLernon *et al.* were developed to predict the chances of cumulative live birth over multiple complete cycles of IVF: a pre-treatment model which predicts cumulative live birth in women before the first complete cycle commences; and a post-treatment model which updates predictions of cumulative live birth after the first fresh embryo transfer episode (McLernon *et al.*, 2016). The models were converted into an online prediction tool called OPIS (Outcome Prediction In Subfertility) (https://w3.abdn.ac.uk/clsm/opis/) and used by 3000 patients and clinicians on average each month. The models which were developed using data from IVF treatments conducted from 1999 to 2008 showed good predictive performance in the development dataset but have not been validated in the UK since. External validation in an independent cohort is essential as it supports the generalizability of the model (Harrell *et al.*, 1996; Steyerberg, 2019). Using prospectively collected Dutch data between 2011 and 2014, a study externally validated the performance of the McLernon *et al.* up to three complete cycles (Leijdekkers *et al.*, 2018). The findings revealed that the pre-treatment model systematically overestimated the probability of cumulative live birth in the external cohort but provided more accurate predictions after recalibration, whilst the post-treatment model calibrated well in the external cohort.

IVF practice in the UK has since undergone major changes, with greater emphasis on elective single embryo transfer and increasing use of frozen-thawed embryo transfers (Human Fertilisation and Embryology Authority, 2018; Ishihara *et al.*, 2014). Therefore, it is important that the McLernon models are externally validated on a more up-to-date cohort of patients to ensure that the predictions are still accurate. Therefore, the aim of the study is to conduct a temporal external validation of the McLernon models in order to demonstrate the continued generalizability of these models to the current UK IVF population.

## Materials and methods

### Data sources

To perform external validation of the McLernon models, this study used the HFEA database which links all fresh and frozen IVF treatment cycles to individual women. Database access was granted following approval by the North of Scotland Research Ethics Committee, the Confidentiality Advisory Group, and the HFEA register research panel. The data were anonymized and transferred to the University of Aberdeen where they were stored on the Data Safe Haven (DaSH) server for analysis.

### Study population

Information was collected from 91 035 women who started their first ovarian stimulation in the UK between January 2010 and December 2016. The records of all complete IVF cycles which began during this period were extracted. Data on frozen embryo transfers associated with these between 1 January 2017 and 31 December 2017 were also collected. No data recorded after the 31 December 2017 were extracted. This data selection method ensured a minimum of 1-year exposure to all embryo transfer attempts within a complete cycle. Women whose treatment involved donor insemination, egg donation and/or surrogacy were excluded.

### Baseline characteristics

For this validation study, the same baseline characteristics that were used in the original McLernon pre- and post-treatment models were selected from the new dataset (with the exception of duration of infertility and pregnancy history which are discussed in the missing data section). The McLernon pre-treatment model predicts the probability of a live birth over six complete cycles at the start of a first complete cycle. Predictions are based on couple characteristics and the type of treatment (IVF or ICSI) to be used. The included predictors are female age (years), duration of infertility (years), causes of infertility (tubal, male factor, anovulation, or unexplained), pregnancy history (yes or no), type of treatment (IVF or ICSI), and treatment year.

After the first fresh embryo transfer, the McLernon post-treatment model revises the predictions using additional treatment-specific data from this cycle. The added predictors are number of eggs collected, availability of cryopreserved embryos, number of embryos transferred (one, two, or three), and stage of transferred embryos, i.e. blastocyst (Day 5 or 6) or cleavage stage (Day 2 or 3). For the validation of the post-treatment model, women who had no eggs collected were excluded as it is impossible for them to achieve a live birth in the first complete cycle.

The number of complete cycles was included in both models as a discrete time variable to predict the probability of a live birth in the *i*th cycle, assuming no live birth occurred in the previous cycle(s). The formulae for calculating the cumulative predicted probability of a live birth over six complete cycles can be seen in Supplementary data files S1 and S2 (McLernon *et al.*, 2016).

### Statistical analysis

#### Missing data: multiple imputation

Data on the duration of infertility were missing for 97% of women, and pregnancy history was entirely missing. This is because the HFEA stopped collecting this information since 2008 (HFEA communication) (Supplementary data file S3). Since these variables were fully recorded from 1999 to 2007, data from this period were used to impute the missing values of these variables in the validation dataset (2010 to 2016).

In the study, three predictor variables had missing values: duration of infertility, pregnancy history, and stage and number of embryos transferred. Multiple imputation of these predictors was performed to increase the statistical power of the model and to adjust for any biases caused by excluding women with missing information (Greenland and Finkle, 1995). Ten imputed datasets were created using the chained equation (MICE) method (to attain a monotone missing data pattern) (Sterne *et al.*, 2009). Then each missing variable was considered as a dependent variable in its own imputation model where it was regressed onto all the other variables. The following variables were included to inform the imputation process: female age, year of treatment, cause of infertility, IVF versus ICSI, and whether embryos were cryopreserved. For the continuous variable ‘duration of infertility’, a predicted mean matching regression model was used; to impute the binary variable ‘pregnancy history’, a logistic regression model was used; and to impute the nominal categorical variable ‘stage and number of embryos transferred’, a discriminant function method was used. This imputation was performed under the assumption that the data were missing at random (MAR) which means that the missing data depend on the values of the observed variables and treatment outcome.

### Model implementation

The predictor values for women in the validation cohort were multiplied by the corresponding parameter estimates of the predictors from the original pre-treatment model and then added together. The same was done for the post-treatment model (McLernon *et al.*, 2016). The predicted probabilities were calculated using the formulas in Supplementary data files S1 and S2.

### Predictive performance

The predictive performance of the McLernon models was evaluated in terms of discrimination and calibration. Discrimination refers to the ability of the models to distinguish between women who will achieve a live birth and those who will not (Moons *et al.*, 2012) and was assessed using the c-statistic.

Calibration refers to the degree of agreement between the observed live birth in the external cohort and predicted live birth (Moons *et al.*, 2012). This was formally assessed using calibration-in-the-large (CIL) and the calibration slope, and graphically assessed using a calibration plot (Cox, 1958; Miller *et al.*, 1993). For perfect calibration, the calibration slope and calibration intercept should be 1 and 0 respectively. We calculated c-statistics, CIL, and calibration slope on each imputed dataset and separate results were pooled using the metamisc package in R version 4.1.1 (Debray *et al.*, 2017). Calibration plots and predicted curves for hypothetical couples were generated using the first imputed dataset. Supplementary data file S4 gives a detailed description of all calibration techniques used in the study.

### Updating the model

Where any model demonstrated poor calibration in the validation cohort, the models were updated using the following three methods to try to improve performance (Steyerberg *et al.*, 2004; Janssen *et al.*, 2008; Moons *et al.*, 2012):

- Update intercept (Method 1): adjustment of the intercept using the calibration intercept;
- Logistic recalibration (Method 2): adjustment of the intercept and the regression coefficients using the calibration intercept and calibration slope; and
- Model revision (Method 3): further model adjustment for individual predictors which had a different effect in the validation cohort compared to the development cohort.

The method which demonstrated the best agreement between the predictions and observed outcomes was used to update each model.

Supplementary data file S5 includes a detailed description of these methods.

All statistical analyses were conducted using STATA version 16 (StataCorp, 2019) and R version 4.1.1 (R Core Team, 2021; Posit team, 2023).

### Patient involvement

No patients were involved in framing the research question, choosing the outcome measures, or developing plans for the design or implementation of the study. Patient input was not sought on interpreting or writing up the results of the study. We have plans to disseminate the results of this research study to patients via national fertility charities and the HFEA.

## Results

Following exclusions, the dataset included 91 035 women who underwent 144 734 complete cycles of IVF/ICSI between January 2010 and December 2016 (Supplementary Fig. S1). The baseline characteristics of couples and the treatments they underwent before initiating IVF are presented in Table 1 for each cohort. The development cohort comprised women who started IVF between 1999 and 2008, whereas the validation cohort consisted of women who started IVF between 2010 and 2016. Women included in the validation cohort had a similar distribution of age to the women in the development cohort. There was also a similar distribution in causes of infertility between the two cohorts.

**Table 1.** Baseline characteristics of couples and their treatment before undergoing the first complete cycle of IVF in the cohorts used for model development and validation.

*Characteristics	HFEA 1999–2008 Development cohort	HFEA 2010–2016 Validation cohort
**Number of patients**	113 873	91 035
**Number of complete cycles**	184 269	137 879
**Patient characteristics**		
**Woman’s age (years)**, Mean (SD)	34.1 (5)	35 (4)
**Duration of infertility (year)**, Median (IQR)		
Complete cases	4 (3–6)	9 (7–12)
Missing, %	18 225 (16)	88 753 (97)^a^
After imputation in validation cohort	–	4 (2–6)
**Pregnancy history**		
No	75 541 (66)	0 (0)
Yes	28 070 (25)	0 (0)
Missing, %	10 262 (9)	91035 (100)
After imputation in validation cohort		
No		57 039 (63)
Yes	–	33 996 (37)^b^
**Causes of infertility**		
Tubal	26 545 (23)	13 493 (15)
Anovulatory	15 942 (14)	11 474 (13)
Male factor	49 753 (44)	35 275 (39)
Unexplained	32 693 (29)	28 433 (31)
Endometriosis	7590 (7)	6709 (7)
More than one	13 414 (12)	10 882 (12)
**Treatment characteristics at completed cycle 1**		
**Type of treatment**		
IVF	67 511 (59)	44 252 (49)
ICSI	46 362 (41)	46 782 (51)
**No of oocytes collected,**		
Median (IQR)	8 (5–13)	9 (6–13)
**No of embryos created,**		
Median (IQR)	5 (2–8)	5 (3–8)
**No of embryos frozen,**		
Median (IQR)	0 (0–1)	0 (0–1)
**Cryopreservation of embryos**	28 950 (25)	31 874 (35)
**Stage and number of embryos transferred**		
Single cleavage	9248 (8)	16 180 (18)
Single blastocyst	662 (1)	27 364 (30)
Double cleavage	75 701 (66)	29 021 (32)
Double Blastocyst	2960 (3)	10 659 (12)
Triple cleavage	8649 (8)	1144 (1)
Triple blastocyst	130 (0.1)	241 (0.3)
No transfer	15 501 (14)	5407 (6)
Missing	1022 (1)	1019 (1)

* The variables listed were included as predictors in the development sample (HFEA 1999–2008 cohort) and the validation sample (HFEA 2010–2017 cohort).

a 93% of women had missing data on duration of infertility in 2010 which increased to almost 100% in 2017.

a,b From 2008, the Human Fertilisation and Embryology Authority (HFEA) changed the layout of their forms for recording data and removed questions regarding pregnancy history and duration of infertility (HFEA communication). Therefore, since these variables were fully recorded from 1998 to 2007, previous pregnancy status (which was 100% missing) and duration of infertility (97% missing) were imputed in the validation dataset using this data to inform the imputation process. IQR: interquartile range.

A higher proportion of women underwent ICSI in the validation cohort compared to women in the development cohort (51% versus 41%). After the first IVF/ICSI cycle, embryo cryopreservation was more frequently performed in women belonging to the validation cohort compared to the development cohort (35% versus 25%). Only 32% of women in the validation cohort had a double cleavage embryo transfer compared to 66% in the development cohort. About half of the women in the validation sample had a single embryo transfer (17.8% had single cleavage-stage transfer and 30.1% had a single blastocyst transfer), whereas only 9% of women in the development dataset had a single embryo transfer.

**Figure 1.**
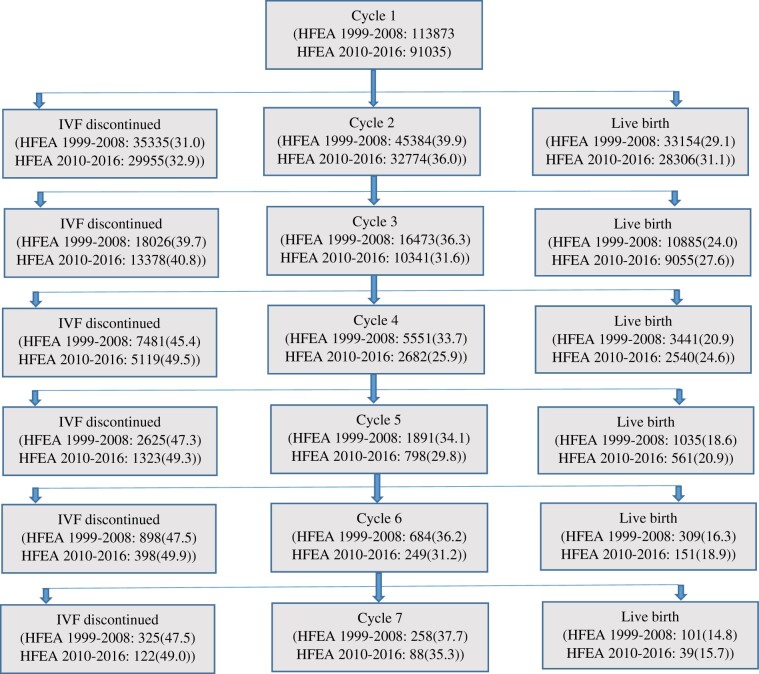
**Flowchart of number of treatments and live birth outcomes over six complete cycles.** Frequency and percentage of women having a live birth, continuing treatment without having a live birth, and discontinuing treatment without having a live birth over six complete cycles of IVF/ICSI in the HFEA 1999–2008 development cohort and 2010–2016 validation cohort. Percentages are in parentheses. HFEA: Human Fertilisation and Embryology Authority.

The number of women in each cohort who started a treatment cycle, had a live birth, or discontinued treatment without having a live birth is presented in Fig. 1. The LBRs per woman were higher in the validation cohort (HFEA 2010–2016) compared to the development cohort (HFEA 1999–2008) for all complete cycles of IVF. Over six complete cycles of IVF/ICSI, the overall LBR of both recent and old HFEA cohorts was 45% and 43%, respectively.

### Predictive performance of the original models

**Figure 2.**
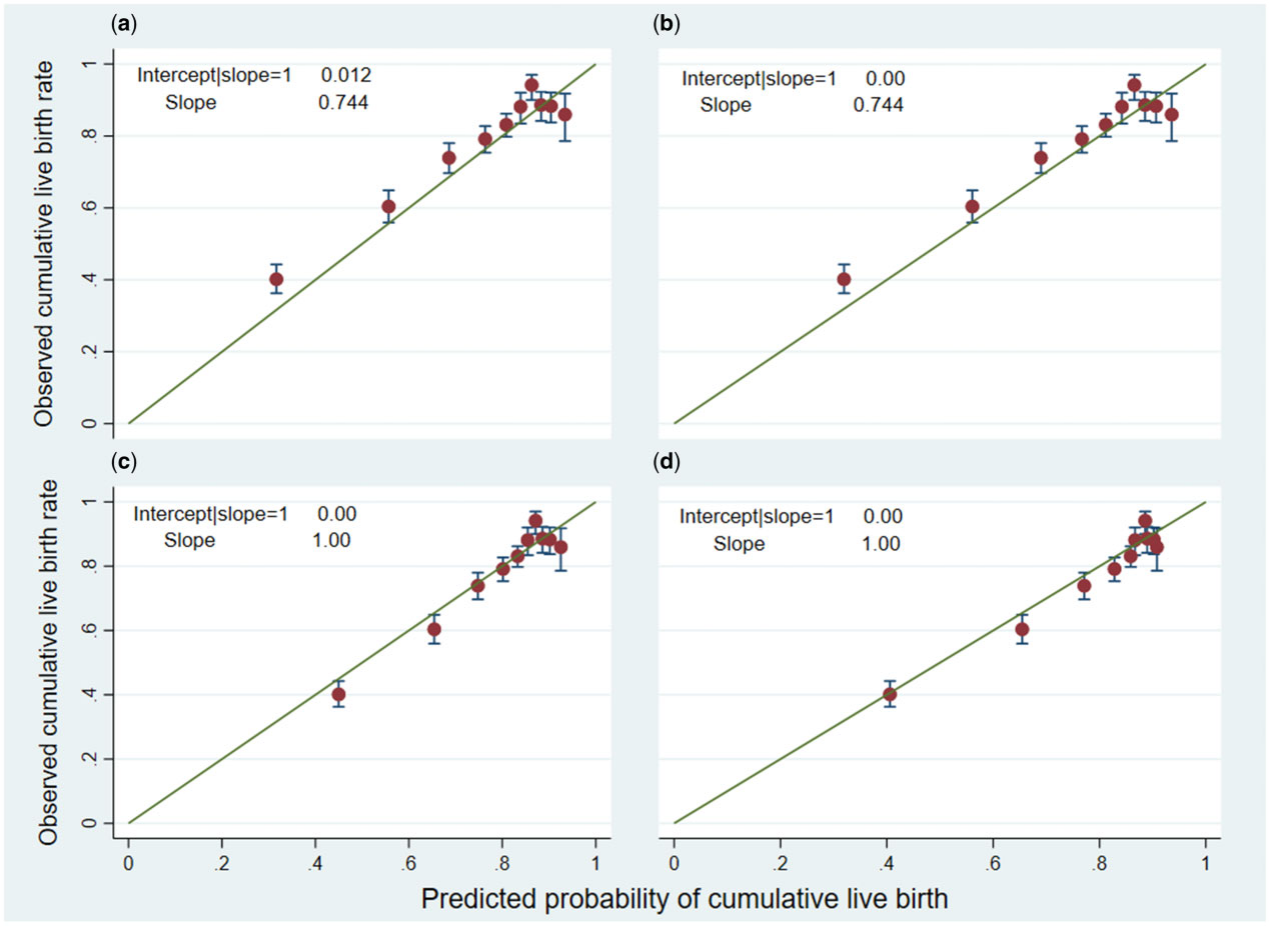
**Calibration plots for the pre-treatment model showing the association between the predicted and observed cumulative live birth rates over six complete IVF/ICSI cycles in the validation dataset.** (**a**) Calibration plot for the original McLernon pre-treatment model as explained by McLernon *et al.* (2016) applied to the validation dataset; (**b**) calibration plot for the recalibrated pre-treatment model following adjustment of the intercept in the validation dataset (update intercept method); (**c**) calibration plot for the recalibrated model following adjustment of both the intercept and slope in the validation dataset (logistic recalibration method 2); and (**d**) calibration plot for the revised model after updating some coefficients using the validation dataset (model revision method).

**Figure 3.**
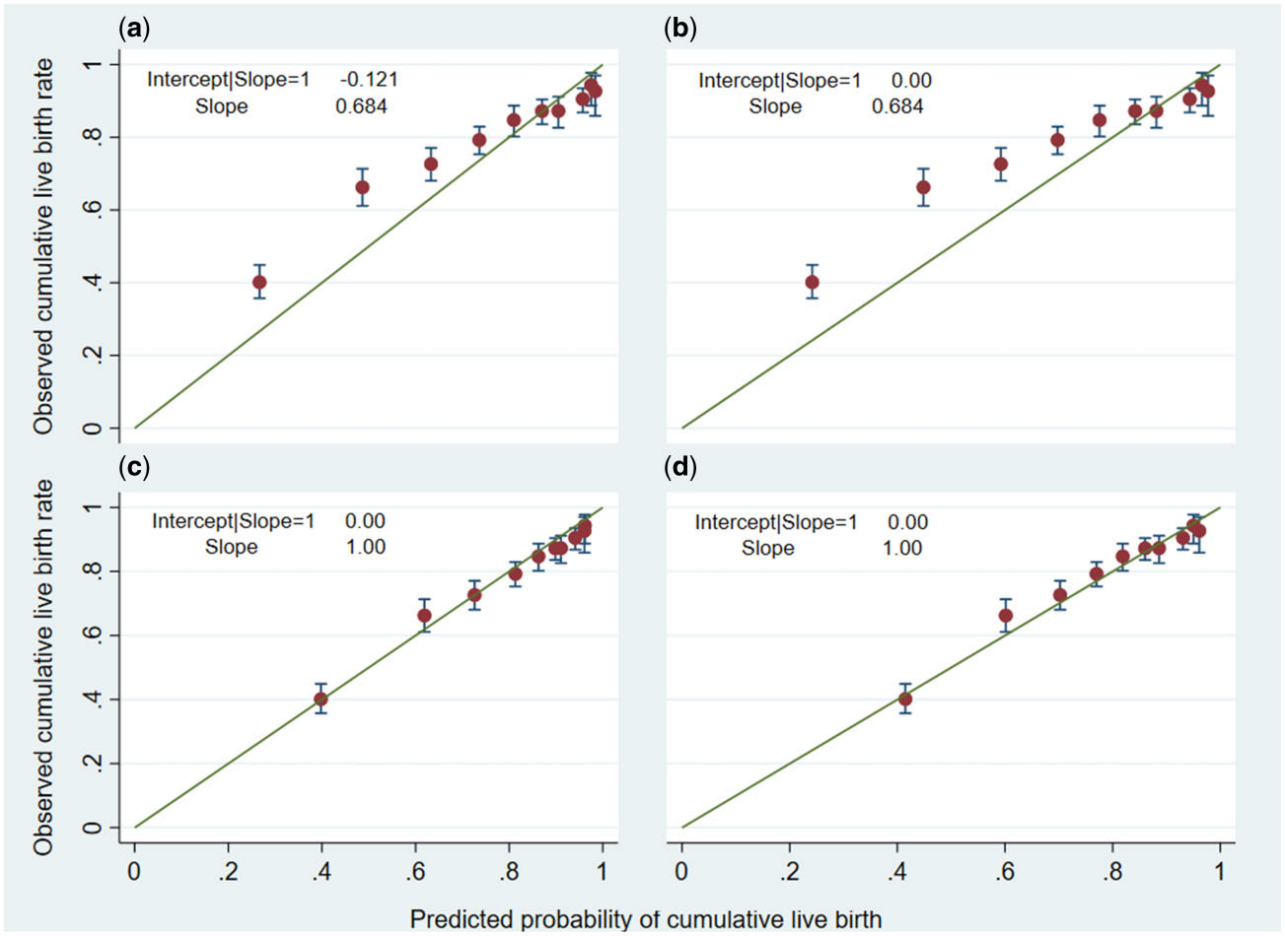
**Calibration plots for the post-treatment model showing the association between the predicted and observed cumulative live birth rates over six complete IVF/ICSI cycles in the validation dataset.** (**a**) Calibration plot for the original McLernon post-treatment model as explained by McLernon *et al.* (2016) applied to the validation dataset; (**b**) calibration plot for the recalibrated post-treatment model following adjustment of the intercept in the validation dataset (update intercept method); (**c**) the recalibrated model following adjustment of both the intercept and slope in the validation dataset (logistic recalibration method); and (**d**) calibration plot for the revised model after updating some coefficients using the validation dataset (model revision method).

In the validation cohort, the pooled c-statistic for the pre-treatment model was 0.68 (95% CI: 0.68 to 0.68) and for the post-treatment model 0.75 (95% CI: 0.75 to 0.75). Figure 2a shows the calibration plot for the first imputed dataset (representative of all 10 imputations) depicting the observed cumulative LBR in the validation cohort versus the predicted probability of cumulative live birth from the pre-treatment model (Fig. 3a shows the post-treatment model calibration plot) (McLernon *et al.*, 2016). The pre-treatment calibration plot had a calibration slope of 0.74 (95% CI: 0.72 to 0.76), and the post-treatment calibration plot had a calibration slope of 0.68 (95% CI: 0.67 to 0.70) (Supplementary Tables S1 and S2). The CIL analyses showed little systematic underestimation for the pre-treatment model (CIL = 0.01 or O/E = 1.01). Systematic overestimation was evidenced for the post-treatment model (CIL = −0.12 or O/E = 0.94) (see the Supplementary Tables S1 and S2). Both the calibration slopes indicated that the original regression coefficient estimates were too large, resulting in extreme predictions in new patients. For example, the calibration slope of 0.74 of the pre-treatment model indicates that the original regression coefficient estimates of the model are over-optimistic by around 26% in new patients, i.e. low chances of live birth calculated by the model are too low and high probabilities are too high compared with the observed LBRs.

Given the poor calibration, both the pre- and post-treatment models were updated in an effort to improve performance in the validation cohort.

### Updating the models

#### The updated pre-treatment model

The estimated parameters of the original pre-treatment model and the three different updated versions of the pre-treatment model (i.e. updated intercept (Method 1), logistic recalibration (Method 2), and model revision (Method 3)) are summarized in Table 2.

**Table 2.** Coefficients of the predictors from the original McLernon pre-treatment model and updated coefficients using three different methods in the validation dataset.

Predictors	Original model	Update intercept (Method 1)	Logistic recalibration (Method 2)	Model revision (Method 3)
**Intercept**	−0.995	−0.983	−1.193	−1.775
**Complete cycle number**				
1 (reference)	0	0	0	0
2	−0.239	−0.239	−0.178	−0.226
3	−0.411	−0.411	−0.306	−0.388
4	−0.563	−0.563	−0.419	−0.531
5	−0.719	−0.719	−0.535	−0.679
6	−0.814	−0.814	−0.606	−0.768
**Couple characteristics**				
**Woman’s age**				
Age	0.028	0.028	0.021	0.025
Age1	−0.181	−0.181	−0.135	−0.222
Age2	0.455	0.455	0.339	0.732
Age3	−1.199	−1.199	−0.892	−1.804
**Duration of infertility, (year)**	−0.029	−0.029	−0.022	−0.016
**Type of treatment,** ICSI versus IVF	0.216	0.216	0.161	−0.006
**Pregnancy history,** no versus yes	−0.077	−0.077	−0.057	−0.143
**Tubal infertility,** yes versus no	−0.096	−0.096	−0.071	−0.091
**Male factor infertility,** yes versus no	−0.101	−0.101	−0.075	0.051
**Anovulatory infertility,** yes versus no	0.049	0.049	0.036	0.139
**Unexplained infertility,** yes versus no	0.060	0.060	0.045	0.057
**Year of first oocyte collection**				
Year	0.033	0.033	0.025	−0.111
Year1	−0.037	−0.037	−0.028	0.255
Year2	0.217	0.217	0.161	−0.587

The estimates of the main parameters of the pre-treatment model using the different updating methods and the details of estimating these parameters are presented in Supplementary Table S3 and Supplementary data file S6, respectively.

The Method 1 updating approach did not lead to any improvement in calibration for the pre-treatment model when reapplied to the validation cohort. However, Methods 2 and 3 did result in improved calibration (Fig. 2b–d). We compared these approaches using Fig. 2 in order to identify the one which had the most beneficial impact on calibration. After recalibrating the original model by adjusting the intercept and slope (Method 2), calibration was good for all tenths except the seventh tenth (as 95% CI of the seventh decile does not overlap with the diagonal reference line) (Fig. 2c). Figure 2d shows a better update of the model (as 95% CIs of all deciles overlap the diagonal reference line) after model revision (Method 3) in the validation cohort and therefore was chosen as the best method to update the pre-treatment model (Supplementary data file S6).

The c-statistic of the model updated by the model revision (Method 3) method decreased very slightly to 0.67 (95% CI: 0.66 to 0.68) (using imputed dataset 1).

#### The updated post-treatment model

The estimated parameters of the original post-treatment model and the three versions of the updated post-treatment model (i.e. updated intercept (Method 1), logistic recalibration (Method 2), and model revision (Method 3)) are summarized in Table 3.

**Table 3.** Coefficients of the predictors from the original McLernon post-treatment model and updated coefficients using three different methods in the validation dataset.

Predictors	Original model	Update intercept (Method 1)	Logistic recalibration (Method 2)	Model revision (Method 3)
**Intercept**	−1.761	−1.882	−2.085	−2.272
**Complete cycle number**				
1 (reference)	0	0	0	0
2	−0.193	−0.193	−0.132	−0.123
3	−0.354	−0.354	−0.242	−0.226
4	−0.512	−0.512	−0.351	−0.327
5	−0.679	−0.679	−0.465	−0.434
6	−0.767	−0.767	−0.525	−0.490
**Couple characteristics**				
**Woman’s age**				
Age	0.027	0.027	0.019	0.028
Age1	−0.156	−0.156	−0.107	−0.213
Age2	0.382	0.382	0.261	0.769
Age3	−1.019	−1.019	−0.697	−1.849
**Duration of infertility,** years	−0.021	−0.021	−0.014	−0.004
**Pregnancy history,** no versus yes	−0.050	−0.050	−0.035	−0.008
**Tubal infertility,** yes versus no	−0.221	−0.221	−0.151	−0.141
**Year of first oocyte collection**				
Year	0.002	0.002	0.001	0.022
Year1	0.062	0.062	0.042	−0.014
**Treatment characteristics at complete cycle 1**				
**Number of oocytes collected**				
Eggs	0.064	0.064	0.044	0.067
Eggs1	−0.050	−0.050	−0.034	−0.061
**Cryopreservation of embryos,** yes vs no	0.650	0.650	0.445	0.517
**Stage and number of embryos transferred**				
Double cleavage stage	0	0	0	0
No embryos transferred	−1.083	−1.083	−0.742	−1.218
Single cleavage stage	−0.566	−0.566	−0.388	−0.404
Single blastocyst stage	0.069	0.069	0.048	0.223
Double Blastocyst stage	0.582	0.582	0.040	0.439
Triple cleavage stage	0.022	0.022	0.015	0.238
Triple blastocyst stage	0.456	0.456	0.312	0.573
Type of treatment, ICSI versus IVF	−0.097	−0.097	−0.066	−0.062

Supplementary Table S4 and Supplementary data file S7 present the different updated estimates of the main post-treatment model parameters and the details of estimating these parameters respectively.

All three updating approaches can be compared to the original model in Fig. 3. In Fig. 3b, some deciles were still outside the diagonal line, indicating that the model updated with Method 1 still needs further improvement. Method 2 (Fig. 3c) showed the best improvement in calibration and was chosen as the best method to update the post-treatment model.

The c-statistic of the model updated by the logistic recalibration (Method 2) method was 0.75 (95% CI: 0.74 to 0.76) (using imputed dataset 1) (Supplementary data file S7).

### Examples of model predictions

**Figure 4.**
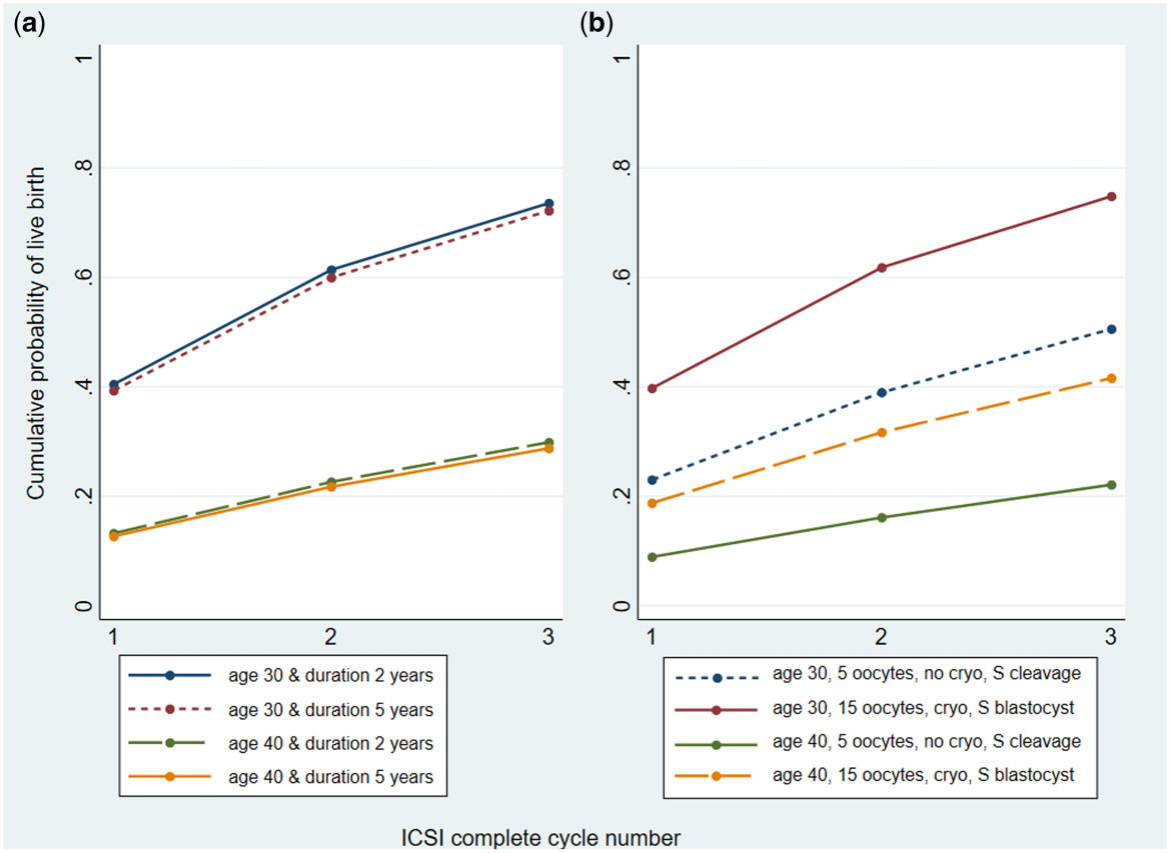
**Examples of the updated models predicting cumulative live birth over three complete cycles of ICSI for couples with different characteristics.** (**a**) couples with either 2 or 5 years of primary male factor infertility, where the female partner is aged either 30 or 40 years (pre-treatment model); (**b**) couples with 2 years of primary male factor infertility, where the female partner is aged either 30 or 40 years, with either 5 or 15 oocytes collected in the first complete cycle. Those with five oocytes have a single cleavage embryo transfer with no embryos cryopreserved, and those with 15 oocytes have a single blastocyst embryo transfer with embryos cryopreserved. S: single.

Figure 4 shows examples of both the pre- and post-treatment model predictions in different case scenarios using the final updated models.

Figure 4a shows the cumulative predictions of live birth from the updated pre-treatment model over three complete ICSI cycles. These are presented for women aged 30 and 40 years with either a 2- or 5-year duration of male factor infertility. As shown in the figure, younger women have a much higher chance of success. A 30-year-old woman with 2 years of infertility has a 41% predicted chance of having a live birth in the first complete ICSI cycle. This increases to 75% over three complete cycles. For a 40-year-old woman with 2 years of primary infertility, these probabilities are 14% and 31% for one complete cycle and three complete cycles, respectively. In contrast, for a similar woman with 5 years of infertility the probabilities are 13% and 30% for one complete cycle and three complete cycles, respectively.

Figure 4b presents the predictions from the updated post-treatment model. The predicted probability of a live birth was updated for a couple with the following characteristics: 30-year-old woman, 2 years of male factor primary infertility, 15 oocytes collected at the start of the first cycle, embryos cryopreserved after fertilization, and a single fresh blastocyst embryo transferred in the first cycle. The predicted probability of live birth after the first complete ICSI cycle is 40%. Cumulatively, this increases to 75% over three complete cycles. A woman who is 40 years old, has five oocytes collected, no embryos cryopreserved, and has a single cleavage stage embryo transferred has a 9% chance of a live birth after the first complete cycle. Cumulatively, this rises to 22% over three complete cycles.

## Discussion

### Main findings

The results of this study show that the pre- and post-treatment models discriminate reasonably well between couples with and without live birth when applied to a more recent cohort of IVF patients. However, both models required updating owing to poor calibration in the external dataset. The updated models should provide more accurate predictions in future patients, and, like the original models, will be incorporated within the OPIS online calculator for regular clinical use.

### Strengths and limitations

For external validation, this study selected the McLernon models which were developed using appropriate methodology, showed good predictive performance ability at both internal and external validation, and scored better on the Transparent Reporting of a multivariable prediction model for Individual Prognosis Or Diagnosis (TRIPOD) checklist than other IVF prediction models (Collins *et al.*, 2015; Ratna *et al.*, 2020).

In the study, calibration was assessed with multiple methods including CIL, logistic calibration, and by visualizing the agreement between the predicted and observed LBRs (Bouwmeester *et al.*, 2012). To improve predictions, the study updated the pre-treatment model using a more extensive model revision method, while the post-treatment model was updated through the simpler approach of recalibration. The recalibration methods (intercept updating and logistic recalibration) are simple and stable because of the low number of parameters estimated. However, the model revision method is expected to lead to a lower bias in the updated model since more parameters are estimated (Steyerberg *et al.*, 2004).

This study has some limitations. First, the external validation exercise involved a dataset with a very high proportion of missing values for duration of infertility (97%) and no data on previous pregnancy. Therefore, both predictors had to be imputed in our analysis. These variables were assumed to be MAR as the missingness is assumed to be conditional on observed variables and treatment outcome. Since these variables were consistently recorded between 1998 and 2007, the patient data from that time period were used to inform the imputation. Multiple, rather than single, imputation was performed as a large amount of missing data may lead to an underestimation of the uncertainty associated with the imputed values (Steyerberg, 2019). Female age explained most of the variation from all of the predictors included in the pre-treatment model, and female age, number of eggs and cryopreservation status explained most in the post-treatment model. However, we cannot rule out the possibility that imputed values for duration of infertility and previous pregnancy could have accounted for some of the difference in model performance in the external cohort compared to the development cohort.

The McLernon models estimate the individualized cumulative chances of live birth under the optimistic assumption that couples who discontinue IVF treatment without a live birth have the same chances of a live birth as couples who continue further treatment cycles. This assumption may lead to an overestimation of the predicted cumulative probability of live birth, as some of the women who discontinue treatment will have stopped because of poor prognosis (Olivius *et al.*, 2004; Brandes *et al.*, 2009) meaning they will have an almost zero chance of conceiving.

The original models were not able to account for other potential predictors, such as BMI, ovarian reserve tests and ethnicity, because they were absent in the HFEA database. We emphasize that the models can only be used in heterosexual couples using their own eggs and sperm and not undergoing preimplantation genetic testing. It should also be noted that the predictions from our models will represent an average prediction over all clinics within the UK. Clinic identifiers are not accessible from the HFEA and so it was not possible to adjust at the individual clinic level.

In October 2009, the HFEA changed their consent policy so that patients had to opt-in for their IVF data to be used for research purposes. Our validation study used data from 2010 and so only would have included couples who opted in. We do not expect there to be a difference in characteristics and outcome between those who opt-in and those who opt-out, but it is difficult to know for sure without access to the data of those who opted out.

We were able to reassess calibration and discrimination after updating both models. This would be considered a type of internal validation as it involves assessing the performance of the updated models in the dataset used to update them. Ideally, we would like to be able to validate the updated models using a dataset from a separate population or to conduct a further temporal validation on a more up to date version of the HFEA dataset. The latter would be preferable from a practical perspective, as the models were developed for, and validated on, UK national data and are intended for use by UK couples. We aim to continue validating the models periodically in the future using UK data to ensure that they remain fit for purpose (Van Calster *et al.*, 2023).

### Interpretation of the findings

Our results show that when applied to more recently treated patients, our models underpredicted outcomes in women with low observed LBRs and slightly overpredicted in women with high observed LBRs. Therefore, it was very important to update these models to reflect current practice and to provide more accurate predictions for patients and clinicians. After updating, the models showed improved agreement between live birth predictions and observed LBRs, as expected. As such, they can be considered suitable for clinical use and can be used to inform future couples of their likely chances of treatment success (Arvis *et al.*, 2012; Zarinara *et al.*, 2016). When updating the McLernon models for the later time period, the differences in the relative weights of the variables was probably a result of a combination of differences in IVF protocols, improved IVF success rates, and differences in case mix between the two cohorts (McLernon *et al.*, 2016; Leijdekkers *et al.*, 2018). The proportion of women having embryo cryopreservation, single embryo transfer, and blastocyst transfer were higher in the validation cohort than the development cohort. This is a result of the increased use of single embryo transfer following the introduction of the UK ‘one-at-a-time’ policy in 2007. It also reflects the increased use of embryo cryopreservation owing to improvements in embryo freezing techniques (Human Fertilisation and Embryology Authority, 2018; Ishihara *et al.*, 2014). These changes in practice and techniques may have resulted in a degree of calibration drift which could explain the different performances of the McLernon models in the validation cohort (Jenkins *et al.*, 2018). Even our updated models will have suffered some calibration drift since the end of our study period in 2016. Since then, the national LBR per embryo transferred has only increased by 1%, from 22% to 23% in 2018, which suggests that not much changed in the following 2 years (Human Fertilisation and Embryology Authority, 2018, 2020). The HFEA has yet to publish data on UK LBRs for 2019–2022 so it is difficult to estimate how much calibration drift has affected our updated model.

Both live birth and treatment discontinuation rates in all complete cycles of IVF were higher in the validation cohort than the development cohort. Year of treatment was strongly positively associated with live birth, reflecting improvements in ART over time (McLernon *et al.*, 2016). From October 2009, the HFEA patient consent forms were changed so that patients had to explicitly agree that their data could be used for research purposes. This change led to higher discontinuation rates owing to many women opting not to disclose their treatment information. Therefore, only couples who provided explicit consent for their information to be used in research were included in this study. Data collected in 2009 were also excluded from this study to ensure that the dataset only encompassed the time period after which the new forms were introduced across the whole of the UK.

Regarding discrimination, the updated pre-treatment model had a slightly lower c-statistic (0.68, 95% CI: 0.67 to 0.69) in the validation cohort than in the development cohort (0.69, 95% CI: 0.68 to 0.69) (McLernon *et al.*, 2018). The recalibrated post-treatment model had a good c-statistic of 0.75 (95% CI: 0.74 to 0.76) in the validation cohort which is slightly lower than the c-statistic of 0.76 (95% CI: 0.75 to 0.77) in the development cohort (McLernon *et al.*, 2018). A previous validation study also reported lower c-statistics of 0.62 (95% Cl: 0.59 to 0.64) and 0.71 (95% CI: 0.69 to 0.74) for the recalibrated pre-treatment McLernon model and the calibrated post-treatment McLernon model, respectively (Leijdekkers *et al.*, 2018). These reductions in model discrimination ability are likely due to the differences in couple and treatment characteristics and outcome prevalence between the two cohorts (Moons *et al.*, 2012).

Poor calibration was evidenced in the external validation for McLernon models. Three increasingly complicated methods were explored for updating the models (i.e. intercept updating, logistic recalibration, and model revision). The method that led to the most improvement (as evidenced by the calibration plot) was selected to update the models (Janssen *et al.*, 2008). Model updating over time is expected given improvements in IVF practice and technology, and changes in patient case mix.

Although our post-treatment model showed good discrimination after recalibration, the discriminatory ability of the pre-treatment model remained reasonably low, as is the case for almost all fertility-based prediction models (Leushuis *et al.*, 2009). The literature suggests that the low c-statistic reflects the homogeneity of the study population e.g. infertile women of reproductive age (Cook, 2008; Coppus *et al.*, 2009). However, a low c-statistic does not necessarily imply that such prediction models have limited use in clinical practice. Couples with a fertility problem are more interested in knowing their chances of live birth (calibration) rather than the ability of the model to discriminate between couples who will have a live birth and couples who will not. Therefore, assessment by calibration is more relevant.

### Comparison with other studies

Two prediction models were developed using national US data from the Society for Assisted Reproductive Technology (SART) (McLernon *et al.*, 2021). The first model is a pre-treatment model, similar to that validated in our current study. The second model is a post-treatment model but differs from the one validated here because it predicts cumulative live birth chances in couples starting a second complete cycle whose first complete cycle was unsuccessful. The pre-treatment model was adjusted for BMI, which was not available for the UK models. Furthermore, anti-Müllerian hormone (AMH) was included in a second pre-treatment model developed using a sub-population who had an AMH measurement. The SART data did not have duration of infertility which was available in the HFEA data and included as a predictor in the UK model. The US models have yet to be externally validated but the c-statistic of the pre-treatment model in the development dataset was slightly higher than that for the UK pre-treatment model (0.71 versus 0.69) (McLernon *et al.*, 2018).

The amount of electronic data produced and stored in the field of reproductive medicine has increased considerably. Artificial intelligence (AI) (or machine learning) is progressively used in medical research to predict future outcomes and is often used in place of regression-based models. Approaches such as Bayesian neural networks and boosting algorithms are more suited to high dimensional datasets, i.e. containing a large number of potential predictors which may include imaging information. Because of this, they require many patients to avoid risk of bias (Andaur Navarro *et al.*, 2021). Models using such approaches that are developed in a single clinic may not be transportable to other clinics as they tend to detect patterns unique to that particular clinic (Chen *et al.*, 2022). However, if clinics are able to share and combine their data to develop such models and then assess heterogeneity in predictive performance between clinics then they may be transportable (Riegler MA *et al.*, 2021). High dimensional electronic health records are not commonly available yet in reproductive medicine (Shingshetty *et al.*, 2022). Our regression-based models will be useful until a reliable and tested AI model has been developed, validated and shown to perform better than our model. There are many publications showing that traditional statistical regression models can match or even outperform AI models (Liew *et al.*, 2022; Lynam *et al.*, 2020). Indeed, statistical models are more generalizable to other populations and easier to interpret.

### Clinical implications

Both the updated models provide more accurate predictions for the current IVF population and can be used as counselling tools in fertility clinics within the UK. Before initiating treatment, the revised pre-treatment model can be used to inform clinicians and couples of their individualized estimates of treatment success over multiple complete cycles of IVF. Then, after the first fresh embryo transfer, the recalibrated post-treatment model can provide a revised estimate of treatment success using treatment-related information. Clinicians can use these models in their daily practice to shape couples’ expectations by informing them of their individualized chances of live birth over a sequence of multiple complete cycles of IVF.

Our models should not be used for excluding couples from treatment. A model which is intended for use in clinical decisions, such as whether or not to have treatment, should be developed using data from patients who were not treated as well as patients who were treated, preferably using data from randomized controlled trials with treated and untreated patients. This would allow us to assess treatment effectiveness (i.e. are couples more likely to have a baby with or without IVF?) and treatment benefit (if they are more likely to have a baby with IVF, is the increase in the predicted chance worth the physical, emotional and financial burden of the treatment?). Our prediction models are not meant to aid decisions around whether to have IVF or ICSI. Such a decision must be made before using the models to make predictions in new patients. For models that aim to facilitate decisions on treatment type, a different causal modelling approach is required when only observational data is available (Sperrin *et al.*, 2019).

The original McLernon models were converted into the OPIS online calculator so that they could be used in clinical practice to estimate the probability of live birth based on the characteristics of the couple and treatment (https://w3.abdn.ac.uk/clsm/opis). Since both the original models underestimate predicted cumulative live birth for couples in the recent UK IVF cohort, conversion of the updated models into a new online calculator is required. The updated online calculator will be able to provide accurate and more up-to-date predictions to both clinicians and couples considering IVF/ICSI treatment.

While we did not involve patients and clinicians in this validation study, our online OPIS calculator has been updated with an optional questionnaire for patients and healthcare professionals to obtain feedback on the tool. We will use the findings to make future refinements to the models and our calculator.

## Conclusion

The updated McLernon prediction models provide accurate predictions of cumulative live birth over multiple complete cycles of treatment which reflect current UK IVF practice. These models, which will be available in our updated OPIS calculator (http://w3.abdn.ac.uk/clsm/opis), can be used as counselling tools to inform couples of their prognosis before commencing IVF/ICSI treatment as well as after the first fresh embryo transfer. They will help couples prepare emotionally and financially for their future treatment.

## Supplementary Material

dead165_Supplementary_data_file_S1

dead165_Supplementary_data_file_S2

dead165_Supplementary_data_file_S3

dead165_Supplementary_data_file_S4

dead165_Supplementary_data_file_S5

dead165_Supplementary_data_file_S6

dead165_Supplementary_data_file_S7

dead165_Supplementary_Figure_S1

dead165_Supplementary_Table_S1

dead165_Supplementary_Table_S2

dead165_Supplementary_Table_S3

dead165_Supplementary_Table_S4

## Data Availability

The data underlying this article cannot be shared publicly due to the privacy of individuals that participated in the study. The data can be shared on reasonable request to the corresponding author with permission of the HFEA. Access to the anonymized HFEA database was approved by the north of Scotland research ethics committee (12/NS/0119), the Confidentiality Advisory Group (CAG), and the HFEA Register Research Panel.
