# Correction to: External validation of models for predicting cumulative live birth over multiple complete cycles of IVF treatment

**DOI:** 10.1093/humrep/deae099

**Published:** 2024-05-10

This is a correction to Mariam B Ratna, Siladitya Bhattacharya, David J McLernon, External validation of models for predicting cumulative live birth over multiple complete cycles of IVF treatment, Human Reproduction, Volume 38, Issue 10, October 2023, Pages 1998-2010, dead165, https://doi.org/10.1093/humrep/dead165

The authors of the above article would like to apologise for accidental errors in the examples included to illustrate pre- and post-treatment modelling of predicted cumulative live birth. The errors occurred because the statistical code for the examples was not updated for the final updated version of the model equation.

In the extended abstract, the last two sentences of the MAIN RESULTS AND THE ROLE OF CHANCE mistakenly state:

*As an example, in the updated pre-treatment model, a 32-year-old woman with 2 years of primary infertility has a 42% chance of having a live birth in the first complete ICSI cycle and a 77% chance over three complete cycles. In a couple with 2 years of primary male factor infertility where a 30-year-old woman has 15 oocytes collected in the first cycle, a single fresh blastocyst embryo transferred in the first cycle and spare embryos cryopreserved, the estimated chance of live birth provided by the post-treatment model is 46% in the first complete ICSI cycle and 81% over three complete cycles.*

It should be:

*As an example, in the updated pre-treatment model, a 30-year-old woman with 2 years of primary infertility has a 41% chance of having a live birth in the first complete ICSI cycle and a 75% chance over three complete cycles. In a couple with 2 years of primary male factor infertility where a 30-year-old woman has 15 oocytes collected in the first cycle, a single fresh blastocyst embryo transferred in the first cycle and spare embryos cryopreserved, the estimated chance of live birth provided by the post-treatment model is 40% in the first complete ICSI cycle and 75% over three complete cycles.*

In the second paragraph of the ‘Examples of model predictions’ section of the ‘Results’, predictions were mistakenly stated as:

*A 30-year-old woman with 2 years of infertility has a 42% predicted chance of having a live birth in the first complete ICSI cycle. This increases to 77% over three complete cycles. For a 40-year-old woman with 2 years of primary infertility, these probabilities are 20% and 45% for one complete cycle and three complete cycles, respectively. In contrast, for a similar woman with 5 years of infertility the probabilities are 19% and 43% for one complete cycle and three complete cycles, respectively.*

It should be:

*A 30-year-old woman with 2 years of infertility has a 41% predicted chance of having a live birth in the first complete ICSI cycle. This increases to 75% over three complete cycles. For a 40-year-old woman with 2 years of primary infertility, these probabilities are 14% and 31% for one complete cycle and three complete cycles, respectively. In contrast, for a similar woman with 5 years of infertility the probabilities are 13% and 30% for one complete cycle and three complete cycles, respectively.*

In the third paragraph of the ‘Examples of model predictions’ section of the ‘Results’, predictions were mistakenly stated as:

*The predicted probability of live birth after the first complete ICSI cycle is 46%. Cumulatively, this increases to 81% over three complete cycles. A woman who is 40 years old, has five oocytes collected, no embryos cryopreserved, and has a single cleavage stage embryo transferred has a 11% chance of a live birth after the first complete cycle. Cumulatively, this rises to 27% over three complete cycles.*

It should be:

*The predicted probability of live birth after the first complete ICSI cycle is 40%. Cumulatively, this increases to 75% over three complete cycles. A woman who is 40 years old, has five oocytes collected, no embryos cryopreserved, and has a single cleavage stage embryo transferred has a 9% chance of a live birth after the first complete cycle. Cumulatively, this rises to 22% over three complete cycles.*

The graphical representation of these prediction modelling characteristics was erroneous in Figure 4. This is correctly illustrated below.

**Figure 4.**
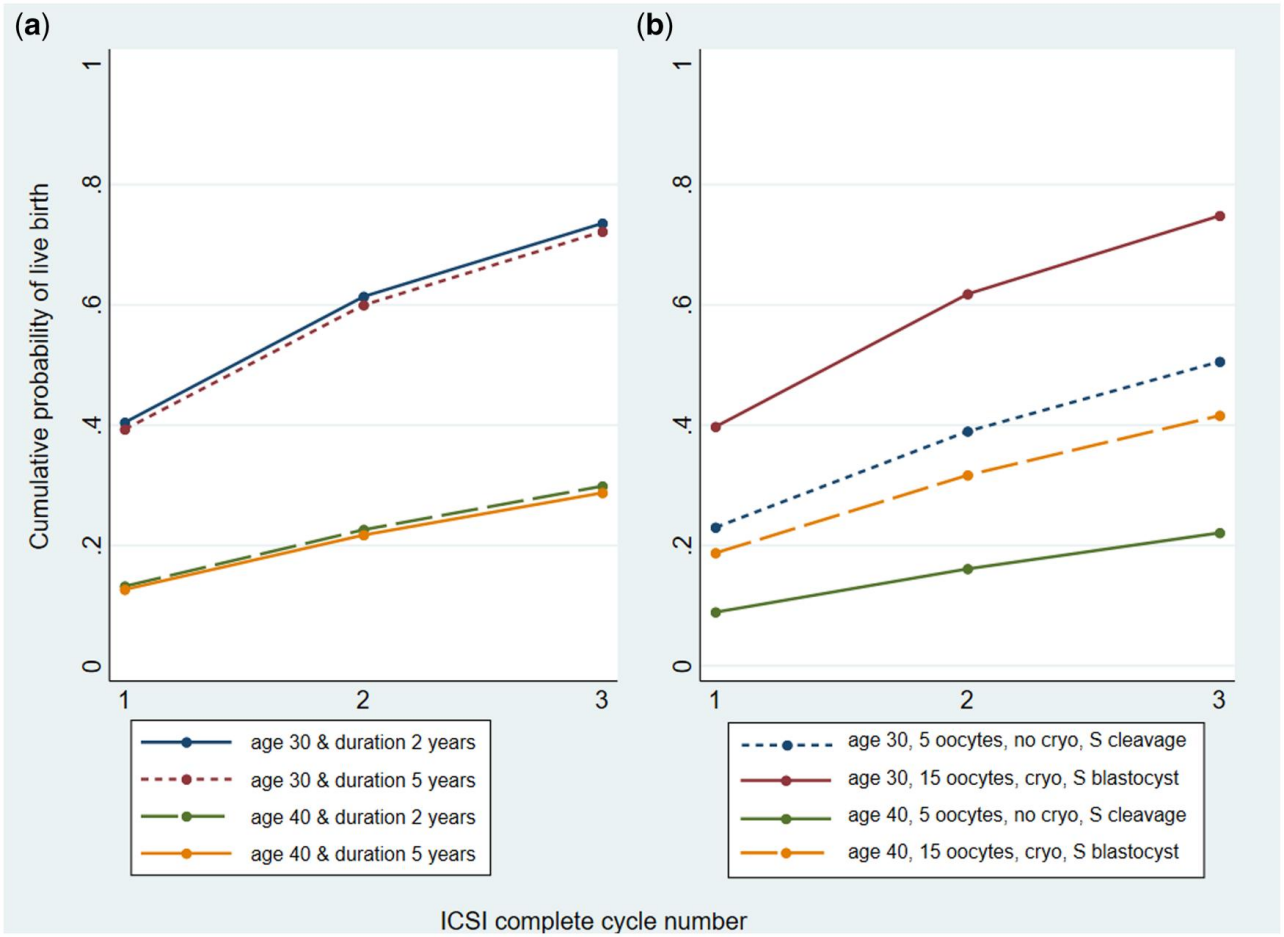
**Examples of the updated models predicting cumulative live birth over three complete cycles of ICSI for couples with different characteristics**. (a) couples with either 2 or 5 years of primary male factor infertility, where the female partner is aged either 30 or 40 years (pretreatment model); (b) couples with 2 years of primary male factor infertility, where the female partner is aged either 30 or 40 years, with either 5 or 15 oocytes collected in the first complete cycle. Those with five oocytes have a single cleavage embryo transfer with no embryos cryopreserved, and those with 15 oocytes have a single blastocyst embryo transfer with embryos cryopreserved. S: single.

The authors would like to assure readers that all other statistical analysis and results are correct. There is no change to the updated model equation or any of the validation results in the paper. The errors are only in the examples given.

The electronic version of this article has been updated at https://doi.org/10.1093/humrep/dead165.

