# Supplementary material for: External validation of models for predicting cumulative live birth over multiple complete cycles of IVF treatment
Source: Hum Reprod. 2023 Aug 25;38(10):1998–2010. doi: 10.1093/humrep/dead165 (PMC10546080; doi:10.1093/humrep/dead165)
Supplement: dead165_Supplementary_data_file_S1 [file dead165_supplementary_data_file_s1.pdf]

OXFORD
human reproduction

Human Reproduction, 2023, 1–1
https://doi.org/10.1093/humrep/dead165
Supplementary Data

Supplementary data file S1

McLernon pre-treatment model.

Text below showing the predictors in the original McLernon pre-treatment model (McLernon *et al.*, 2016).

Predictor name, description, and range of possible values:

Age—Female age (18 to 50 years)
Duration—How long have you been trying to conceive? (0 to 21 years)
Previous—Have you been pregnant before? (1 = No; 0 = Yes)
Tubal—Do you have a problem with your tubes? (1 = Yes; 0 = No)
Anovulation—Do you have an ovulation problem? (1 = Yes; 0 = No)
MaleFactor—Do you have a male factor fertility problem? (1 = Yes; 0 = No)
Unexplained—Do you have an unexplained fertility problem? (1 = Yes; 0 = No)
Treatment—Which fertility treatment are you planning on having? (1 = ICSI; 0 = IVF)

Original pre-treatment formulas for calculating the predicted probability of live birth after the first, second, …., sixth cycle of IVF are as follows:

1. For the non-linear association between Age and the probability of a live birth, the following Age1 to Age3 equations are first calculated and then put in the XB equation below (Point 3).
   - Age1 = max((Age − 26)/k,0)**3 + (11*max((Age − 41)/k,0)**3 − (15)*max((Age − 37)/k,0)**3)/4;
   - Age2 = max((Age − 31)/k,0)**3 + (6*max((Age − 41)/k,0)**3 − (10)*max((Age − 37)/k,0)**3)/4;
   - Age3 = max((Age − 34)/k,0)**3 + (3*max((Age − 41)/k,0)**3 − (7)*max((Age − 37)/k,0)**3)/4; k = 15**(2/3); **means 'to the power of'
2. For the non-linear association between Year and the probability of a live birth, the following Year1 and Year2 equations are first calculated and then put in the XB equation below (Point 3). The value Year = 0 is used for the most up to date predictions.
   - Year1 = max((Year + 9)/k,0)**3 + ((6)*max((Year)/k,0)**3 − (9)*max((Year + 3)/k,0)**3)/(3);
   - Year2 = max((Year + 6)/k,0)**3 + ((3)*max((Year)/k,0)**3 − (6)*max((Year + 3)/k,0)**3)/(3); k = 9**(2/3)
3. Calculate XB
   XB = −0.9948 + 0.0362[#] + (0.0275*Age) + (−0.1805*Age1) + (0.4553*Age2) + (−1.1990*Age3) + (−0.0295*Duration) + (−0.0772*Previous) + (−0.0957*Tubal) + (0.0492*Anovulation) + (−0.1005*MaleFactor) + (0.0602*Unexplained) + (0.2155*Treatment) + (0.0334*Year) + (−0.0370*Year1) + (0.2173*Year2)
   [#]To inflate predictions to 2013 an additional 0.0362 is added
4. PCycle1 = exp (XB)/(1 + exp (XB))
   PCycle2 = exp (XB − 0.2394)/(1 + exp (XB − 0.2394))
   PCycle3 = exp (XB − 0.4110)/(1 + exp (XB − 0.4110))
   PCycle4 = exp (XB − 0.5628)/(1 + exp (XB − 0.5628))
   PCycle5 = exp (XB − 0.7189)/(1 + exp (XB − 0.7189))
   PCycle6 = exp (XB − 0.8138)/(1 + exp (XB − 0.8138))
5. To calculate the cumulative probabilities of a live birth after first, second, ……, sixth cycles we will use the following formulas:
   CumPCycle1 = 1 − (1 − PCycle1)
   CumPCycle2 = 1 − (1 − PCycle1) * (1 − PCycle2)
   CumPCycle3 = 1 − (1 − PCycle1) * (1 − PCycle2) * (1 − PCycle3)
   CumPCycle4 = 1 − (1 − PCycle1) * (1 − PCycle2) * (1 − PCycle3) * (1 − PCycle4)
   CumPCycle5 = 1 − (1 − PCycle1) * (1 − PCycle2) * (1 − PCycle3) * (1 − PCycle4) * (1 − PCycle5)
   CumPCycle6 = 1 − (1 − PCycle1) * (1 − PCycle2) * (1 − PCycle3) * (1 − PCycle4) * (1 − PCycle5) * (1 − PCycle6)
