# Supplementary material for: External validation of models for predicting cumulative live birth over multiple complete cycles of IVF treatment
Source: Hum Reprod. 2023 Aug 25;38(10):1998–2010. doi: 10.1093/humrep/dead165 (PMC10546080; doi:10.1093/humrep/dead165)
Supplement: dead165_Supplementary_data_file_S2 [file dead165_supplementary_data_file_s2.pdf]

OXFORD
human reproduction

Human Reproduction, 2023, 1–1
https://doi.org/10.1093/humrep/dead165
Supplementary Data

Supplementary data file S2

McLernon post-treatment model.

Text below showing the predictors in the original McLernon post-treatment model (McLernon *et al.*, 2016).

Predictor name, description, and range of possible values:

Age—Female age (18 to 50 years)
Duration—How long have you been trying to conceive? (0 to 21 years)
Previous—Have you been pregnant before? (1 = No; 0 = Yes)
Tubal—Do you have a problem with your tubes? (1 = Yes; 0 = No)
Eggs—How many eggs were collected on your first IVF cycle? (1 to 28)
Treat—Was your first cycle IVF or ICSI? (1 = ICSI; 0 = IVF)
Cryo—In your first cycle did you have embryos frozen? (1 = Yes; 0 = No)

Stage—What type of embryo transfer did you have in your first fresh embryo transfer? (No embryos transferred; single cleavage stage; single blastocyst stage; double cleavage stage; double blastocyst stage; triple cleavage stage; triple blastocyst stage)

1. For the non-linear association between Age and the probability of a live birth, the following Age1 to Age3 equations are first calculated and then put in the XB equation below (Point 4).
   - Age1 = max((Age − 26)/k,0)**3 + (11*max((Age − 41)/k,0)**3 − (15)*max((Age − 37)/k,0)**3)/4;
   - Age2 = max((Age − 31)/k,0)**3 + (6*max((Age − 41)/k,0)**3 − (10)*max((Age − 37)/k,0)**3)/4;
   - Age3 = max((Age − 34)/k,0)**3 + (3*max((Age − 41)/k,0)**3 − (7)*max((Age − 37)/k,0)**3)/4; k = 15**(2/3); **means ‘to the power of’
2. For the non-linear association between Year and the probability of a live birth, the following Year1 and Year2 equations are first calculated and then put in the XB equation below (Point 4). The value Year = 0 is used for the most up to date predictions.
   - Year1 = max((Year + 8)/k,0)**3 + ((4)*max((Year + 1)/k,0)**3 − (7)*max((Year + 4)/k,0)**3)/(3); k = 7**(2/3
3. For the non-linear association between Eggs and the probability of a live birth, the following Eggs1 equation is first calculated and then put in the XB equation below (Point 4)
   - Eggs1 = max((Eggs − 3)/k,0)**3+((6)*max((Eggs − 18)/k,0)**3 − (15)*max((Eggs − 9)/k,0)**3)/(9); k = 15**(2/3)
4. Calculate XB
   XB = −1.7564 + 0.0362[‡] + (0.0272*Age) + (−0.1556*Age1) + (0.3812*Age2) + (−1.0184*Age3) + (−0.0208*Duration) + (−0.0504*Previous) + (−0.2207*Tubal) + (0.0018*Year) + (0.0619*Year1) + (0.0630*Eggs) + (−0.0479*Eggs1) + (−0.0968*Treat) + (0.6490*Cryo) + Stage[§]
   [‡]To inflate predictions to 2013 an additional 0.0362 is added
   [§]Stage equals the following values depending on group chosen:
   If double cleavage stage then Stage = 0;
   If no embryos transferred then Stage = −1.0842;
   If single cleavage stage then Stage = −0.5675;
   If single blastocyst stage then Stage = 0.0684;
   If double blastocyst stage then Stage = 0.5802;
   If triple cleavage stage then Stage = 0.0218;
   If triple blastocyst stage then Stage = 0.4547
5. Therefore, formulas for calculating the probabilities of live birth after the first, second, . . ., sixth cycle of IVF are as follows:
   PCycle1 = exp (XB)/(1 + exp (XB))
   PCycle2 = exp (XB − 0.1933)/(1 + exp (XB − 0.1933))
   PCycle3 = exp (XB − 0.3537)/(1 + exp (XB − 0.3537))
   PCycle4 = exp (XB − 0.5122)/(1 + exp (XB − 0.5122))
   PCycle5 = exp (XB − 0.6788)/(1 + exp (XB − 0.6788))
   PCycle6 = exp (XB − 0.7666)/(1 + exp (XB − 0.7666))
6. To calculate the cumulative probabilities of a live birth after first, second, ..., sixth cycles we will use the following formulas:
   CumPCycle1 = 1 − (1 − PCycle1)
   CumPCycle2 = 1 − (1 − PCycle1) * (1 − PCycle2)
   CumPCycle3 = 1 − (1 − PCycle1) * (1 − PCycle2) * (1 − PCycle3)
   CumPCycle4 = 1 − (1 − PCycle1) * (1 − PCycle2) * (1 − PCycle3) * (1 − PCycle4)
   CumPCycle5 = 1 − (1 − PCycle1) * (1 − PCycle2) * (1 − PCycle3) * (1 − PCycle4) * (1 − PCycle5)
   CumPCycle6 = 1 − (1 − PCycle1) * (1 − PCycle2) * (1 − PCycle3) * (1 − PCycle4) * (1 − PCycle5) * (1 − PCycle6)
