# Supplementary material for: External validation of models for predicting cumulative live birth over multiple complete cycles of IVF treatment
Source: Hum Reprod. 2023 Aug 25;38(10):1998–2010. doi: 10.1093/humrep/dead165 (PMC10546080; doi:10.1093/humrep/dead165)
Supplement: dead165_Supplementary_data_file_S3 [file dead165_supplementary_data_file_s3.pdf]

OXFORD
human reproduction
Human Reproduction, 2023, 1–1
https://doi.org/10.1093/humrep/dead165
Supplementary Data

Supplementary data file S3

HFEA communication

Currently, HFEA uses four different forms to collect information from all licensed fertility clinics across the UK. These include a patient and partner registration form, IVF treatment form, early outcome form, and outcome form. Patients and partners are registered once at each clinic where they undergo treatment. These treatments may have an early outcome reported on the corresponding form, following which, the final outcome is also recorded. The number of forms and the information recorded on them have changed over the years. Prior to 2007, patients and their partners were registered on the same form (rather than two separate forms) and the early outcome information was recorded on the treatment form. Prior to 2000, registration and treatment data were supplied on the same form. After October 2007, along with the introduction of electronic forms and online validation, some further changes were made on the forms. These included the removal of records regarding the patient and their partners' pregnancy history and the duration of time in which the couples spent trying to get pregnant.
