# Supplementary material for: External validation of models for predicting cumulative live birth over multiple complete cycles of IVF treatment
Source: Hum Reprod. 2023 Aug 25;38(10):1998–2010. doi: 10.1093/humrep/dead165 (PMC10546080; doi:10.1093/humrep/dead165)
Supplement: dead165_Supplementary_data_file_S4 [file dead165_supplementary_data_file_s4.pdf]

OXFORD
human reproduction
Human Reproduction, 2023, 1–1
https://doi.org/10.1093/humrep/dead165
Supplementary Data

Supplementary data file S4

Calibration at external validation

To calculate the calibration slope, the linear predictor (i.e. the sum of the multiplication between all predictor values and their corresponding coefficients from the original model) was used as an explanatory variable in a logistic regression model. The parameter estimate of the linear predictor is known as the calibration slope. For perfect calibration, the calibration slope and calibration intercept should be 1 and 0, respectively. A calibration slope of <1 means that the regression coefficients of the original model have led to extreme predictions in the validation cohort (i.e. low predictions are too low and high predictions are too high). On the other hand, a calibration slope of more than 1 indicates that the regression coefficients of the original model were too close to 0.

As it is difficult to interpret the calibration intercept when the calibration slope is not equal to 1, for the estimation of the calibration intercept the calibration slope was fixed at 1. This is known as calibration-in-the-large (also known as mean calibration) and shows the difference between the mean predicted probability and the observed probability. A calibration-in-the-large which is different from 0 indicates that the model's predicted probabilities in the validation cohort are systematically too high/overestimated (intercept < 0) or too low/underestimated (intercept > 0). Such a difference in the calibration intercept commonly reflects a difference in outcome incidence between the development cohort and the validation cohort which cannot be explained by different distributions of the predictor values. For this study, calibration-in-the-large was calculated by fitting the logistic regression model, with the regression coefficient of the linear predictor fixed at 1 (i.e. included as an offset term). Another approach for assessing calibration-in-the-large is to use the ratio of the proportion of observed events in the external dataset to the average predicted probability after applying the model to the external dataset (called O/E).

For the creation of the calibration plot, patients were given a ranking based on their predicted probabilities from the first complete cycle, and then were arranged into ten subgroups in the validation cohort. Within the subgroups, the Kaplan–Meier estimates of the observed cumulative probability of live birth over six complete cycles were plotted against the mean predicted cumulative probability of live birth. All plotted points were then compared against a diagonal regression line with a slope of 1 and an intercept of 0 representing perfect calibration (Cox, 1958). To note, points plotted below this diagonal line indicate an overestimation of the predicted probability of live birth, whilst points plotted above it indicate an underestimation.
