# Supplementary material for: External validation of models for predicting cumulative live birth over multiple complete cycles of IVF treatment
Source: Hum Reprod. 2023 Aug 25;38(10):1998–2010. doi: 10.1093/humrep/dead165 (PMC10546080; doi:10.1093/humrep/dead165)
Supplement: dead165_Supplementary_data_file_S5 [file dead165_supplementary_data_file_s5.pdf]

OXFORD
human reproduction

Human Reproduction, 2023, 1–1
https://doi.org/10.1093/humrep/dead165
Supplementary Data

Supplementary data file S5

Updating the model

To overcome poor calibration-in-the-large, Method 1 was used. Here, the mean predicted probability is equal to the observed outcome frequency (Steyerberg *et al.*, 2004). In this method, only the intercept of the original model was updated. This was achieved by fitting a logistic regression model where the linear predictor of the original model was applied to the validation dataset as an offset (see Equation (1)).

$$\ln\left(\frac{P(\text{livebirth})}{1 - P(\text{livebirth})}\right) = \alpha_{\text{calibration}} + \text{linear predictor(offset)} \quad (1)$$

For Method 2 (also known as the logistic recalibration method), both the calibration intercept and calibration slope were updated. To do this, a logistic regression model was fitted to the validation dataset, with the linear predictor from the original model applied to the external dataset as the only covariate (see Equation (2)).

$$ln\left(\frac{P(\text{livebirth})}{1 - P(\text{livebirth})}\right) = \alpha_{\text{calibration}} + \beta_{\text{calibration}} \times \text{linear predictor} \quad (2)$$

When the value of the calibration slope, $\beta_{\text{calibration}}$, is equal to 1 this indicates that the original regression coefficients do not need adjustment and that there is no overfitting. However, as the calibration slope, $\beta_{\text{calibration}}$, was significantly different from 1, this method was used to recalibrate the original regression coefficients i.e. the regression coefficients of the original models were multiplied by the calibration slope. The intercept of the original models was also adjusted by adding the calibration intercept, $\alpha_{\text{calibration}}$.

When predictor effects of a model are heterogeneous between the development and validation cohorts and calibration plots of these two cohorts show different predictions across the whole range of predicted probabilities, then the logistic recalibration method will not improve model fit. Instead, Method 3, a more extensive updating method, known as model revision, must be used to re-estimate the regression coefficients of some or all predictors. It may also include some additional predictors (Cox 1958; van Houwelingen, 2000). A logistic regression model of Equation (3) was therefore fitted to the validation dataset, adjusting for the linear predictor from the original model applied to the new patients and each predictor of the original model.

$$ln\left(\frac{P(\text{livebirth})}{1 - P(\text{livebirth})}\right) = \alpha_{\text{calibration}} + \beta_{\text{calibration}} \times \text{linear predictor} + \gamma \times \text{predictor} \quad (3)$$

where, γ is the deviation from the recalibration coefficient value (based on the recalibration method). When the value of γ is significantly different to 0, this indicates that the effect of the predictor is still different in the validation sample after performing the recalibration method. The likelihood ratio test ($P < 0.05$) was then performed to test whether the deviation had added predictive value. This process was repeated with all the predictors using a forward stepwise approach, starting with the predictor which had the largest Wald statistic for γ (Steyerberg *et al.*, 2004; Steyerberg, 2019).
