# Supplementary material for: External validation of models for predicting cumulative live birth over multiple complete cycles of IVF treatment
Source: Hum Reprod. 2023 Aug 25;38(10):1998–2010. doi: 10.1093/humrep/dead165 (PMC10546080; doi:10.1093/humrep/dead165)
Supplement: dead165_Supplementary_data_file_S6 [file dead165_supplementary_data_file_s6.pdf]

OXFORD
human reproduction
Human Reproduction, 2023, 1–1
https://doi.org/10.1093/humrep/dead165
Supplementary Data

Supplementary data file S6

Pre-treatment model updating details

Only the intercept of the original model was updated in Method 1. Corresponding with the low predicted probabilities in the validation cohort, the intercept of the updated model was increased by adding the constant (0.012) from the calibration-in-the-large assessment. Using Method 2 (adjusting the intercept and the regression coefficients of the original model by the calibration intercept and calibration slope, respectively), the individual regression coefficients were multiplied by 0.744 and the intercept of the original pre-treatment model was decreased by 0.197. The updated regression coefficients were estimated by multiplying the regression coefficients of the original pre-treatment model with the calibration slope of Method 2. For example, for male factor infertility this resulted in an updated regression coefficient of [−0.101 (original regression coefficient) * 0.744 (calibration slope)] = −0.075.

Method 3 (re-estimation of some of the predictors with a different association between the validation cohort and the development cohort) showed statistically different effects of predictors including age, year of treatment, type of treatment, male factor infertility, duration of infertility, anovulatory infertility, and pregnancy history on live birth between the validation cohort and the development cohort ($P < 0.001$). The effects of the other predictors (unexplained and tubal infertility) on live birth were not significantly different between the cohorts. For example, the updated regression coefficient for male factor infertility was estimated by multiplying the regression coefficient of this predictor from the original pre-treatment model (−0.101) with the calibration slope (0.944). The deviation from this regression coefficient (0.146; see Supplementary Table S3) was then added. This resulted in 0.146 + (−0.101 * 0.944) = 0.051 becoming the updated regression coefficient for male factor infertility.

When the model was updated using Methods 1 and 2 the c-statistic was the same as for original pre-treatment model applied to the validation cohort (c-statistic: 0.68, 95% CI: 0.67 to 0.69). This is because only a constant term was added in Method 1, and all coefficients were multiplied by the same number in Method 2. Hence, the rank order of predictions (which is used to calculate the c-statistic) remained the same (Janssen *et al.*, 2008).
