# Supplementary material for: External validation of models for predicting cumulative live birth over multiple complete cycles of IVF treatment
Source: Hum Reprod. 2023 Aug 25;38(10):1998–2010. doi: 10.1093/humrep/dead165 (PMC10546080; doi:10.1093/humrep/dead165)
Supplement: dead165_Supplementary_data_file_S7 [file dead165_supplementary_data_file_s7.pdf]

OXFORD
human reproduction
Human Reproduction, 2023, 1–1
https://doi.org/10.1093/humrep/dead165
Supplementary Data

Supplementary data file S7

Post-treatment model updating details

In Method 1, only the intercept of the original model was updated. The intercept of the updated model was decreased by 0.121 corresponding with the low predicted probabilities in the validation cohort. In Method 2, the updated regression coefficients were estimated by multiplying the regression coefficients of the original post-treatment model with the calibration slope of Method 2, 0.684. The intercept was also decreased by subtracting 0.324.

The results from Method 3 showed that there were statistically significant differences in the effects of predictors such as year of treatment, stage and number of embryos transferred, age, duration of infertility, number of oocytes collected, cryopreservation of embryos, and pregnancy history on live birth between the validation and the development cohorts ($P < 0.001$). The effects of the other predictors (tubal infertility and type of treatment) were not statistically significantly different between the cohorts.

After model revision using Method 3, the c-statistic of the model decreased slightly to 0.74 (0.73 to 0.75).
