# Supplementary material for: External validation of models for predicting cumulative live birth over multiple complete cycles of IVF treatment
Source: Hum Reprod. 2023 Aug 25;38(10):1998–2010. doi: 10.1093/humrep/dead165 (PMC10546080; doi:10.1093/humrep/dead165)
Supplement: dead165_Supplementary_Figure_S1 [file dead165_supplementary_figure_s1.pdf]

Human Reproduction, 2023, 1–1
https://doi.org/10.1093/humrep/dead165
Supplementary Data

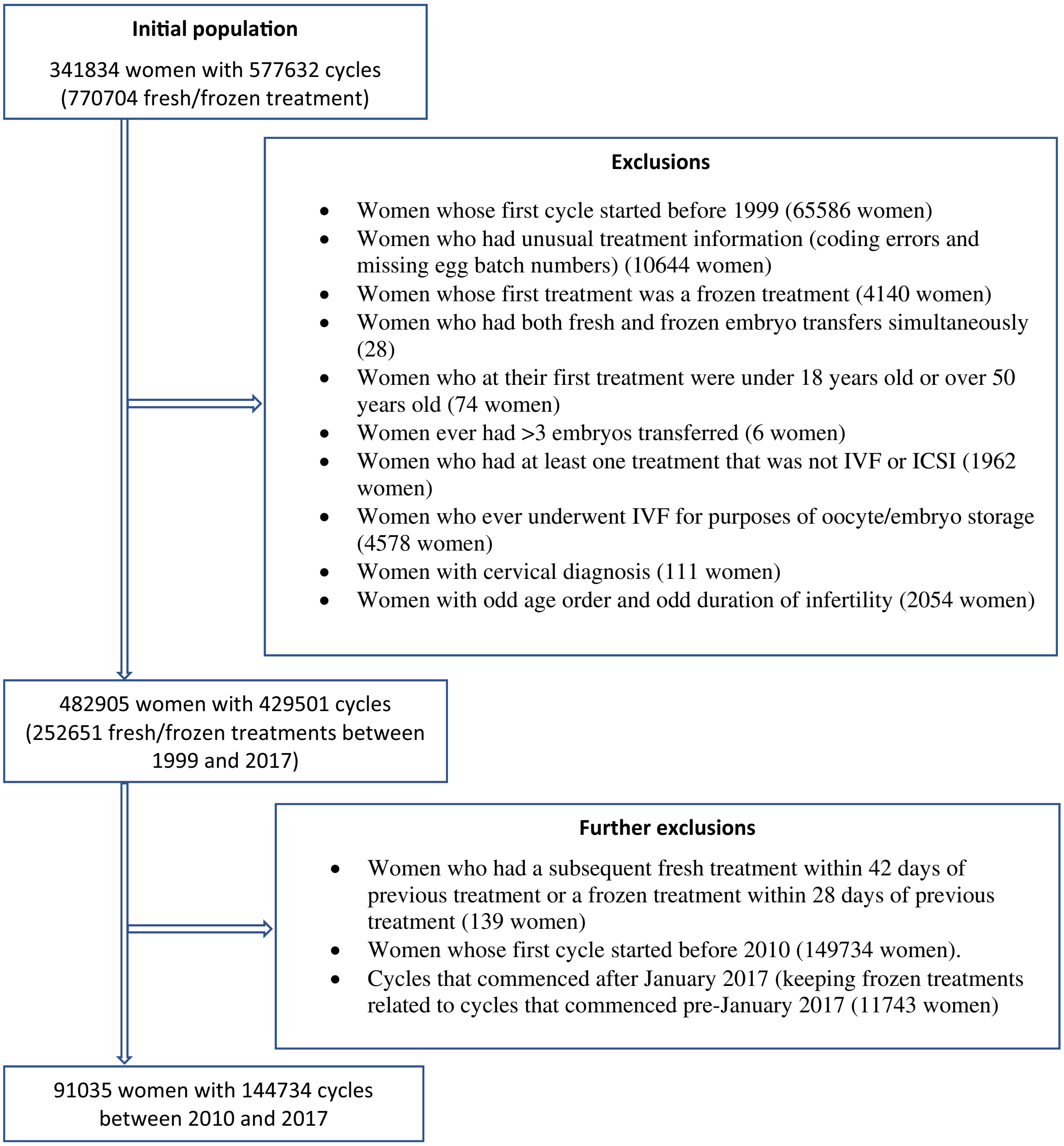

Supplementary Figure S1. Flow chart of exclusion criteria for the temporal validation of the McLernon pre- and post-treatment models.
