# Supplementary material for: External validation of models for predicting cumulative live birth over multiple complete cycles of IVF treatment
Source: Hum Reprod. 2023 Aug 25;38(10):1998–2010. doi: 10.1093/humrep/dead165 (PMC10546080; doi:10.1093/humrep/dead165)
Supplement: dead165_Supplementary_Table_S1 [file dead165_supplementary_table_s1.pdf]

OXFORD
human reproduction

Human Reproduction, 2023, 1–1
https://doi.org/10.1093/humrep/dead165
Supplementary Data

Supplementary Table S1. c-statistic, calibration-in-the-large, and calibration slope of the pre-treatment model in the validation sample for 10 imputed datasets.

Imputed datasets	c-statistic (95% CI)	Calibration-in-the-large	O/E	Calibration slope (95% CI)
**Dataset 1**	0.679 (0.667 to 0.690)	0.012	1.007	0.744 ((0.723 to 0.765)
**Dataset 2**	0.678 (0.667 to 0.689)	0.016	1.009	0.741 (0.720 to 0.762)
**Dataset 3**	0.677 (0.666 to 0.688)	0.015	1.008	0.741 (0.720 to 0.762)
**Dataset 4**	0.679 (0.668 to 0.690)	0.012	1.007	0.748 (0.727 to 0.769)
**Dataset 5**	0.678 (0.667 to 0.689)	0.015	1.008	0.744 (0.723 to 0.765)
**Dataset 6**	0.679 (0.668 to 0.690)	0.012	1.007	0.747 (0.726 to 0.769)
**Dataset 7**	0.678 (0.667 to 0.689)	0.015	1.008	0.744 (0.723 to 0.766)
**Dataset 8**	0.677 (0.666 to 0.688)	0.015	1.008	0.741 (0.720 to 0.762)
**Dataset 9**	0.677 (0.666 to 0.688)	0.014	1.008	0.743 (0.722 to 0.764)
**Dataset 10**	0.677 (0.666 to 0.688)	0.016	1.009	0.747 (0.725 to 0.768)
**Pooled**	**0.678 (0.677 to 0.679)**	**0.014**	**1.008 (1.007 to 1.009)**	**0.744 (0.742 to 0.746)**

Before pooling the C-statistic, we applied a logit transformation as described in Debray *et al.* (2017). We used the *valmeta* function of the *metamisc* package in R version 4.1.1. A similar approach was used for pooling calibration slope. For O/E, we applied a log transformation as described in the same article by Debray *et al.* (2017).

Debray TPA, Damen JAAG, Snell KIE, Ensor J, Hooft L, Reitsma JB, Riley RD, Moons KGM. A guide to systematic review and meta-analysis of prediction model performance. BMJ 2017;356:i6460.
