# Supplementary material for: External validation of models for predicting cumulative live birth over multiple complete cycles of IVF treatment
Source: Hum Reprod. 2023 Aug 25;38(10):1998–2010. doi: 10.1093/humrep/dead165 (PMC10546080; doi:10.1093/humrep/dead165)
Supplement: dead165_Supplementary_Table_S2 [file dead165_supplementary_table_s2.pdf]

Human Reproduction, 2023, 1–1
https://doi.org/10.1093/humrep/dead165
Supplementary Data

Supplementary Table S2. c-statistic, calibration-in-the-large, and calibration slope of the post-treatment model in the validation sample for 10 imputed datasets.

Imputed datasets	c-statistic (95% CI)	Calibration-in-the-large	O/E	Calibration slope (95% CI)
**Dataset 1**	0.754 (0.743 to 0.764)	−0.121	0.937	0.684 (0.669 to 0.698)
**Dataset 2**	0.754 (0.743 to 0.764)	−0.118	0.938	0.685 (0.671 to 0.700)
**Dataset 3**	0.753 (0.743 to 0.763)	−0.119	0.938	0.685 (0.671 to 0.699)
**Dataset 4**	0.754 (0.743 to 0.764)	−0.121	0.937	0.685 (0.670 to 0.699)
**Dataset 5**	0.754 (0.743 to 0.764)	−0.119	0.938	0.685 (0.671 to 0.700)
**Dataset 6**	0.754 (0.743 to 0.764)	−0.122	0.936	0.685 (0.670 to 0.699)
**Dataset 7**	0.754 (0.743 to 0.764)	−0.119	0.938	0.685 (0.671 to 0.700)
**Dataset 8**	0.754 (0.743 to 0.764)	−0.118	0.938	0.685 (0.670 to 0.700)
**Dataset 9**	0.753 (0.743 to 0.764)	−0.119	0.938	0.684 (0.670 to 0.699)
**Dataset 10**	0.754 (0.743 to 0.764)	−0.118	0.938	0.687 (0.672 to 0.701)
**Pooled**	**0.754 (0.753 to 0.754)**	**−0.119**	**0.938 (0.937 to 0.938)**	**0.685 (0.684 to 0.686)**

Before pooling the C-statistic, we applied a logit transformation as described in Debray *et al.* (2017). We used the *valmeta* function of the *metamisc* package in R version 4.1.1. A similar approach was used for pooling calibration slope. For O/E, we applied a log transformation as described in the same article by Debray *et al.* (2017).

Debray TPA, Damen JAAG, Snell KIE, Ensor J, Hooft L, Reitsma JB, Riley RD, Moons KGM. A guide to systematic review and meta-analysis of prediction model performance. BMJ 2017;356:i6460.
