# Supplementary material for: External validation of models for predicting cumulative live birth over multiple complete cycles of IVF treatment
Source: Hum Reprod. 2023 Aug 25;38(10):1998–2010. doi: 10.1093/humrep/dead165 (PMC10546080; doi:10.1093/humrep/dead165)
Supplement: dead165_Supplementary_Table_S3 [file dead165_supplementary_table_s3.pdf]

OXFORD

human reproduction

Human Reproduction, 2023, 1–1
https://doi.org/10.1093/humrep/dead165
Supplementary Data

Supplementary Table S3. Data on updates of the McLernon pre-treatment model in the validation cohort according to the update intercept method, logistic recalibration method, and model revision method with statistically significant changes to coefficients.

Predictors	Update intercept (Method 1)	Logistic recalibration (Method 2)	Model revision (Method 3)
**Calibration intercept**	0.012[a]	−0.197	−0.780
**Calibration slope**	–	0.744	0.944
**Woman's age**			
Age	–	–	0.001
Age1			−0.051
Age2			0.303
Age3			−0.672
**Year of first oocyte collection**			
Year	–	–	−0.142
Year1			0.290
Year2			−0.792
**Type of treatment**			
ICSI vs IVF	–	–	−0.210
**Male factor**	–	–	0.146
**Duration of infertility, (year)**	–	–	0.011
**Anovulation**	–	–	0.092
**Pregnancy history**			
No vs yes	–	–	−0.070

[a] Calibration intercept with calibration slope fixed at 1.
