# Supplementary material for: External validation of models for predicting cumulative live birth over multiple complete cycles of IVF treatment
Source: Hum Reprod. 2023 Aug 25;38(10):1998–2010. doi: 10.1093/humrep/dead165 (PMC10546080; doi:10.1093/humrep/dead165)
Supplement: dead165_Supplementary_Table_S4 [file dead165_supplementary_table_s4.pdf]

OXFORD
human reproduction

Human Reproduction, 2023, 1–1
https://doi.org/10.1093/humrep/dead165
Supplementary Data

Supplementary Table S4. Data on updates of the McLernon post-treatment model in the validation cohort according to the update intercept method, logistic recalibration method, and model revision method with statistically significant changes to coefficients.

Predictors	Update intercept (Method 1)	Logistic recalibration (Method 2)	Model revision (Method 3)
**Calibration intercept**	−0.121[a]	−0.324	−0.511
**Calibration slope**	–	0.684	0.639
**Year of first oocyte collection**			
Year			0.021
Year1	–	–	−0.054
**Stage and number of embryos transferred**			
Double cleavage stage			0
No embryos transferred	–	–	−0.526
Single cleavage stage			−0.043
Single blastocyst stage			0.179
Double blastocyst stage			0.067
Triple cleavage stage			0.223
Triple blastocyst stage			0.282
**Woman's age**			
Age	–	–	0.011
Age1			−0.113
Age2			0.525
Age3			−1.198
**Duration of infertility, years**	–	–	0.010
**Number of oocytes collected**			
Eggs			0.026
Eggs1	–	–	−0.029
**Cryopreservation of embryos,** yes vs no	–	–	0.102
**Pregnancy history,** no vs yes	–	–	0.024

[a] Calibration intercept with calibration slope fixed at 1.
